# Physical Activity Protects the Human Brain against Metabolic Stress Induced by a Postprandial and Chronic Inflammation

**DOI:** 10.1155/2015/569869

**Published:** 2015-05-05

**Authors:** Leo Pruimboom, Charles L. Raison, Frits A. J. Muskiet

**Affiliations:** ^1^Natura Foundation, Edisonstraat 66, 3281 NC Numansdorp, Netherlands; ^2^Department of Laboratory Medicine, University Medical Center Groningen (UMCG), University of Groningen, P.O. Box 30.001, 9700 RB Groningen, Netherlands; ^3^Department of Psychiatry, College of Medicine and Norton School of Family and Consumer Sciences, College of Agriculture and Life Sciences, University of Arizona, Tucson, AZ, USA

## Abstract

In recent years, it has become clear that chronic systemic low-grade inflammation is at the root of many, if not all, typically Western diseases associated with the metabolic syndrome. While much focus has been given to sedentary lifestyle as a cause of chronic inflammation, it is less often appreciated that chronic inflammation may also promote a sedentary lifestyle, which in turn causes chronic inflammation. Given that even minor increases in chronic inflammation reduce brain volume in otherwise healthy individuals, the bidirectional relationship between inflammation and sedentary behaviour may explain why humans have lost brain volume in the last 30,000 years and also intelligence in the last 30 years. We review evidence that lack of physical activity induces chronic low-grade inflammation and, consequently, an energy conflict between the selfish immune system and the selfish brain. Although the notion that increased physical activity would improve health in the modern world is widespread, here we provide a novel perspective on this truism by providing evidence that recovery of normal human behaviour, such as spontaneous physical activity, would calm proinflammatory activity, thereby allocating more energy to the brain and other organs, and by doing so would improve human health.

## 1. Introduction

Chronic inflammatory diseases are a major cause of morbidity and impaired work and social functioning and are responsible for 35 million to 52 million annual deaths worldwide (WHO 2014) [1]. A state of low-grade inflammation might be considered the “cause of causes” for these deadly and disparate conditions. In contrast to inflammatory patterns observed in hunter-gatherer groups living more in accord with lifestyles that were prototypical across human evolution and in which inflammatory responses are brisk and time-limited (i.e., resolving within a maximum of 42 days), in the modern world chronic proinflammatory activity can last for weeks, months, or even years [2]. Many of the inflammatory diseases that plague modern societies were uncommon until some 200 years ago [3] but are nowadays increasingly prevalent. However, treatment is still in its infancy [4], and interventions addressing the genuine etiologies of the diseases have typically been less than fully satisfactory [5].

Although the abnormalities that promote allergy, asthma, autoimmunity, and other more systemic inflammatory states, such as cardiovascular disease, are usually characterized in terms of their immune effects, it is less appreciated that the underlying activation of both innate and adaptive immune inflammatory pathways has costly consequences in terms of resource use of energy, proteins, and minerals such as calcium [6] and magnesium [7–9]. In addition, chronic activation of the immune system produces a constant flux of energy and blood to the immune system itself, which leads to metabolic stress on other organs and, in certain circumstances, also the brain. Stress on the brain is unexpected in light of the brain's selfish character and humans' high encephalization quotient, which is the highest of all mammals and provides evidence of how evolution prioritized energy allocation to the human brain [10]. Thus, as we discuss in this paper, these metabolic and nutrient imbalances have wide ranging effects on health-relevant physiological functioning that go beyond the straightforward costs of immune activation* per se*.

One of the major risk factors for chronic low-grade activity of the immune system is frequent and abundant food intake. Postprandial inflammation can put a high burden on energy availability for the whole body, including the brain, as shown by the typical “post-lunch dip” in energy and mental activity observed after high-fat and high-glycemic-load meals [11]. Postprandial inflammation increases with meal size, meal frequency, and consumption of foul food, and these factors reduce brain growth in mammals, illustrating the metabolic conflict between the immune system and the brain [12].

Our ancestors experienced rapid and consistent brain growth for 2.5 million years, despite the likelihood that they frequently consumed spoiled food in the absence of food preservation technology [13]. This pattern of rapid and sustained brain growth despite consumptive patterns known to activate postprandial inflammation suggests that other mechanisms inhibited chronic inflammation—and by extension also attenuated postprandial immune activation. While a number of factors may have contributed to this inhibition, in this review we will focus on evidence suggesting that preprandial physical activity attenuates postprandial inflammatory activity while simultaneously protecting from infection via the induction of immunoglobulins, including lysozyme, and especially, lactoferrin.

In human adults, lactoferrin is produced during and after exercise independent of sex, age, and menstrual cycle [14]. In infants, lactoferrin is obtained through breast milk, consumed 6 to 7 times daily by newborns. Newborns allocate 74% of their energy intake to brain growth and differentiation [15–17]; an energy allocation that would be impossible if they experienced a high-cost bout of postprandial inflammation after every meal. Fortunately, constituents found in breast milk are capable of downregulating the newborn's immune system, while they also protect against possible pathogens. Lactoferrin is probably the most abundant immunologically active constituent of breast milk that reaches concentrations as high as 8 mg/g in colostrum and 1.5 to 4 mg/g in mature milk [18]. A breastfed newborn who consumes 600 mL of breast milk daily can ingest up to 2.4 grams of lactoferrin daily, an amount that is probably more than enough to protect against infection, while simultaneously inhibiting postprandial inflammatory activity of adipocytes and the immune system, thereby saving energy for constant brain growth.

The hominin brain experienced unprecedented growth despite the challenges that the immune system faced as our ancestors sought novel experiences and colonized the world. However, in the last 30,000 years, brain size has shrunk from 1,490 mL to 1,350 mL, a loss of 11% [19]. Here we suggest that this reduction in brain size happened because of new environmental inflammatory factors such as novel pathogens, high meal frequency, food abundance, and lack of solar radiation but most of all because of the absence of regular physical activity.

## 2. Energy and Energy Conflict as the Driving Force behind Evolution

In all animals, energy conflicts shaped physiology during evolution. These conflicts seem to have caused changes in energy allocation among organs. Nowhere have these conflicts been more relevant than in the rapid expansion of the brain during hominin evolution [20, 21]. In principle, a proportionally larger and metabolically expensive brain could have been supported through an increase in the basal metabolic rate, but no evidence of such a relationship has been found in humans or other primates [22]. Because of this, several ideas have been advanced to explain how this larger brain might have been metabolically supported. For example, the expensive tissue hypothesis [20] and, more recently, various energy-allocation scenarios [16, 22] propose that the development of the metabolically expensive human brain must have had consequences for other organs, leading to lower energy allocation to those organs in favour of the brain.

Navarrete et al. [23] examined evidence for the expensive tissue hypothesis in 100 different mammalian species, including 23 primates. They found that, controlling for fat-free body mass, brain size is not inversely correlated with the mass of the digestive tract or any other expensive organ, thus refuting the expensive tissue hypothesis. However, they did find evidence for a negative correlation between brain size and fat depots in mammals, raising the intriguing possibility that encephalization and fat storage both evolved as compensatory strategies to buffer against starvation. When these two strategies are combined, fat might provide extra energy for the brain during starvation, but only if this fat storage did not negatively impact locomotor efficiency. Central fat storage (which does not hamper locomotion efficiency) has probably favoured encephalization, redirecting energy allocation from growth, reproduction, and high-energy locomotion to brain development and more efficient locomotion, including during starvation [23].

The observation that human bipedal locomotion and foot anatomy lead to less energy use than the bipedal or quadrupedal locomotion of chimpanzees is supporting this theory [24]. Bipedal locomotion in humans also facilitates higher central fat depots without major energy demands during movement [23]. A recent study provides new evidence for the expensive tissue hypothesis [25]. The study shows that guppies with a bigger brain have smaller guts and produce fewer offspring. This is in line with the expensive tissue hypothesis and adds a second factor to this hypothesis relating brain size, gut length, and reproductive capacity [25]. Humans are “under-muscled” compared with other primates, although this difference is too small to provide sufficient energy for brain growth [22]. Nevertheless, it is the energy consumed by human muscles during locomotion which is almost twice as low compared with, for instance, in chimpanzees (332 versus 564 kcal/day) [22] providing enough energy for brain development and function. The consequence is that humans' brains are three times bigger than the brains of chimpanzees, and brain metabolism accounts for 25% of the basal metabolic rate in humans and only 7 to 8% in other primate species [26]. Overall, there seems to be sufficient scientific support to suggest that the increase of human brain size and metabolism has been possible because of a change of locomotion, higher central fat depot storage [27], and (although not addressed in this review) a change of food intake (see review [24]). In addition, the human brain may have benefitted from a change in the expression of glucose transporters [28] and the same holds for the immune system. Higher expression of GLUT1, supporting high and constant glucose uptake, is seen in activated immune cells, where energy is needed to protect against pathogens and other immune challenges [29]. This occurs at the expense of GLUT4 that needs insulin for glucose uptake and is notably expressed in muscles and adipocytes [30–33]. Thus, immune function may have benefited from a smaller gut, reduced energy needs for locomotion, increased fat mass, and tissue-specific differences in the expression of glucose transporters.

The work of Fedrigo et al. [28] provides a new explanation for this intriguing “mystery,” that is, the evidence that during human evolution energy allocation has been directed to brain and immune system development. They showed that human brain cells express more activity of the SLC2A1 gene responsible for the production of GLUT1 glucose transporters compared with the chimpanzee and macaque (human > chimpanzee > macaque). At the same time, SLC2A4 expression (i.e., GLUT4) in muscle is significantly higher in chimpanzee > human > macaque. Given a certain circulating glucose concentration, an increase in the amount of SLC2A1 or SLC2A4 protein per gram tissue results in more glucose being captured by that tissue and less by others. It is important to note that GLUT1 transporters are insulin-independent whereas GLUT4 in muscles depends on insulin for glucose uptake. An increase in SLC2A1 expression in the brain and a decrease in SLC2A4 in skeletal muscle would work synergistically to change the distribution of glucose between these two most energy-demanding tissues in the human body, in a manner allowing more energy to become available for the brain.

## 3. Glucose to the Immune System: Prioritizing Energy Guidance

A similar line of reasoning explains glucose allocation to the immune system. The energy demands of lymphocytes and leukocytes increase dramatically upon activation [3, 5] and all activated immune cells express GLUT1 glucose transporters [29, 34]. Not surprisingly, several signals and stimuli such as pathogen associated molecular patterns (PAMP), including lipopolysaccharide (LPS), can promote GLUT1 expression, giving rise to an increase of insulin-independent glucose transport into all immune cells that supports T-cell receptor stimulation, immune cell migration, and inflammation [35, 36]. Thus, higher expression of GLUT1 promotes energy allocation to the immune system, which could be considered an “energy demand reaction,” mobilizing fuel stocks and suppressing the resource demand of other organs/systems with the overall purpose of preferentially allocating fuels to activated immune cells [37]. Glucose allocation to the immune system maintains its function even under strong energy restriction [38].

Metabolic mechanisms and immune control coevolved, conceivably because both processes are interrelated and essential to survival [39], and both seem to have originated in a single fat body organ, as can still be seen in* Drosophila melanogaster* [40, 41]. The integration of metabolism and immunology persists in higher organisms, in which lymph nodes are embedded in perinodal adipose tissue that may influence immune responses. In humans, adipose tissue is well infiltrated with macrophages, and the production of inflammatory cytokines by both adipocytes and macrophages contributes to systemic inflammation [42].

Activation of the immune system through danger signals utilizes and redistributes energy in a manner that favours the brain and the immune system. However, prolonged activation of the immune system, as observed in people with chronic inflammatory states, allocates glucose chronically to the immune system through immune-controlled downregulation of GLUT1 transporters at the level of the blood-brain barrier and a reduction of GLUT4 transporters at the level of muscle and adipose tissue [43–45]. This selfish behaviour of the immune system is responsible for the majority of chronic diseases, if not all [46]. Our hypothesis is that chronic exercise reallocates energy primarily to the brain and muscles, reducing energy distribution to the immune system, and by doing so recovers metabolic homeostasis.

## 4. Muscle as a Defence Mechanism in Humans

Muscle is a “forgotten” organ of the immune system. Indeed, the physical activity permitted by muscles changes the phenotype of immune function from proinflammatory to anti-inflammatory, while maintaining protection against possible lethal pathogens. Interestingly, chronic exercise is only seen in humans and allowed us to discover and inhabit widely divergent habitats around the world, while remaining able to survive and reproduce. Hominins in general and humans in particular tended to seek novelty in their environments, and this practice would have led them to encounter new pathogens, climatological challenges, and food scarcity, all which would have threatened survival and prompted activation of the immune system. If proinflammatory activity had dominated the biology of our ancestors when they were challenged with pathogens such as* Plasmodium falciparum* malaria, tuberculosis,* Salmonella*, or pathogenic* Escherichia coli*, brain growth from 450 mL in early* Homo erectus* to the current 1,350 mL in modern humans would unlikely have been possible. The energy costs of a consistently active immune system would have been enormous and would probably have impeded the growth of the brain as well as the body. An overly active immune system would also have harmed reproduction [47], as evidenced in modern fertility issues among autoimmune patients [48].

Hominins are not the only organisms in which such a trade-off occurs. Green plants and hominins probably share one and the same ancestor [49]. The difference is that plants have chosen an evolutionary path in which locomotion had low or no priority, which implies that they have not learned to escape from danger in a physical manner [50]. Animals' ability to physically flee prevents them from being wounded and thereby from experiencing the ensuing cytotoxic and self-damaging reaction of the highly expensive innate immune system [51]. Without the means to physically escape from predators or other direct threats, plants employ toxins to defend themselves against predators. These toxins {e.g., perforin} are defence substances similar to those used by animals to kill invading pathogens and are* part of the immune system* of the plant [52]. Plants do not have the “migrating” immune cells found in animals and are therefore dependent on an immune system in each individual cell [53], which has the potential to be very robust. As a result, in plants a chronic pathogenic load can lead to overactivity in their equivalent of an immune system causing a change in energy distribution that favours immune functions at the expense of functions related to growth and reproduction [54].

Similar to plants, humans and other mammals may experience damage following a robust reaction of their immune system, such as in sepsis. Long-term and very intense activation of the immune system in individuals with sepsis can cause loss of lean body mass, tissue breakdown for use as a source of amino acids and energy, growth impairment, and reproductive disturbances [48, 55]. Even motor and mental functions in later life may be affected [55, 56]. In addition, a robust inflammatory reaction following acute trauma, such as a stroke or any other neurotrauma, often produces severe secondary damage [57]. The strength of the immune reaction results from the complexity of the human immune system, which has high receptor diversity that greatly enhances the efficiency of microbial detection. However, these same qualities also make humans uniquely vulnerable to autoimmune disease (AID) [58], as high receptor diversity increases the risk of misinterpretation of self-molecules as foreign, potentially provoking autoimmune responses [59].

### 4.1. The Sedentary Death Syndrome: The Immune System Takes Over

The prevalence of autoimmunity is markedly increased in sedentary people [60], who, by definition, infrequently engage in physical activity. It is interesting to consider whether the expensive immune reaction seen in sedentary individuals can be considered adaptive in a manner similar to the strategy plants use to defend against invaders and danger, given that—like plants—sedentary individuals are less able than others to flee danger.

Exercise has powerful immune effects. Even nonstrenuous exercise, when engaged in regularly, promotes better health and increases life expectancy [61]. Nonstrenuous exercise also induces the expression of anti-inflammatory molecules such as lactoferrin and lysozymes, which provide defence against a large number of invaders, including bacteria, viruses, and other microbes [62]. A bout of forty-five minutes of running at 75% of VO2 max increases the production of lysozyme and lactoferrin in saliva significantly, independent of sex and menstrual phase [14]. Serum lactoferrin concentrations also increase immediately after strenuous exercise, such as running, and may play an antibacterial role in host defences prior to the mobilization of neutrophils into the circulating pool, thereby attenuating the need for a possible inflammatory response with high-energy demand [63, 64]. Sedentary individuals, obese people, and patients with diabetes mellitus type 2 exhibit lower lactoferrin levels [65, 66], while suffering from an energy-demanding, chronic low-grade inflammation [10].

All organisms share a need for food intake, and food intake produces postprandial immune activation as a result of the possible presence of danger signals. This immune reaction is normally based on the production of inflammation-preventing defence molecules such as lactoferrin, immunoglobulin A (IgA), and lysozyme, which prevent the high-energy demands incurred from activation of innate immune cells [65]. However, when this mechanism fails or is inadequate, as often happens in obese/sedentary individuals, an inflammatory response ensues [67]. Increasing evidence suggests that this type of postprandial inflammatory response may contribute to the development of metabolic syndrome, endothelial dysfunction, cardiovascular diseases, obesity, insulin resistance, and chronic low-grade inflammation [60, 65].

Just as postprandial inflammation is more common in those who are sedentary or obese, postprandial inflammation is also observed after consumption of a meal that is high in fat, consists of refined carbohydrates, or contains sugar, fructose, linoleic acid, or other high-caloric nutrients. Interestingly, a postprandial inflammatory reaction may occur after an even modestly sized meal that contains cereal-fed meat which should be considered “new” on an evolutionary time scale [68–70]. The same holds for consumption of a new form of hybridized beef. The postprandial immune response after ingestion of “new” wagyu beef is significantly higher than when humans ingest “old” kangaroo meat [70]. All these data raise the intriguing possibility that the increased immune activity against new nutrients reflects a “borrowed” ancient immune reaction against evolutionary unknown danger signals, causing low-grade, systemic, inflammation [70, 71]. Another factor increasing inflammatory activity of the immune system is chronic food availability. The relationship between food availability and development of the metabolic syndrome has been investigated in mice [72, 73] and more recently in humans. Hatori et al. [72] showed that obesity and metabolic syndrome are caused not only by caloric content, but also by food being constantly available. Koopman et al. [73] showed that meal frequency impacts development of fatty liver. Using a 40% hypercaloric diet, they showed that a high meal frequency (6/day) as compared to a lower frequency (3/day) increases intrahepatic triglycerides and abdominal fat, independent of caloric content and body weight gain. Constant food availability further decreases overall physical activity and especially spontaneous physical activity [74]. In contrast, starvation upregulates glucose transport through the blood-brain barrier by increasing the number of insulin-independent glucose transporters 1 (GLUT1) [75], whereas starvation leads to spontaneous physical activity and even hyperactivity [76].

## 5. Exercise Protects the Body against the Expensive and Damaging Postprandial Inflammatory Response: Our Hypothesis and Its Putative Mechanism

Interestingly, exercise prior to meal consumption markedly lowers the postprandial inflammatory reaction [77]. Evidence suggests that this effect may result from muscle contraction inducing anti-inflammatory myocytokines (i.e., myokines), including IL-6, IL-15, and IL-8, that subsequently stimulate production and release of anti-inflammatory molecules such as lysozyme and lactoferrin by the immune system [64, 78]. The previously mentioned anti-inflammatory myokines are increased up to 100-fold during exercise [79] and thereby are responsible for the production of anti-inflammatory molecules such as lactoferrin during and after physical activity [80]. Elevated concentrations of lactoferrin in serum have been reported 30 minutes after intense running and are associated with elevated serum antibacterial activity to viable* Micrococcus luteus* [62]. Furthermore, higher levels of serum lactoferrin have also been observed two hours after submaximal cycling followed by a bout of eccentric resistance exercise [81]. The lactoferrin increase appears to be related to intense exercise and eccentric contraction, which has also been observed in relation to the production of anti-inflammatory myokines [82].

There seems to be a direct relationship between glycogen depletion and the production of muscle-derived IL-6, which, despite its inflammatory activities, also demonstrates anti-inflammatory and metabolic-sensing and regulating properties when expressed as a muscle myokine [82]. Lactoferrin stimulates the expression of a protein in adipocytes and in newly identified cells of the innate immune system called six-transmembrane protein of prostate 2 (STAMP-2) [83]. STAMP-2 links obesity, inflammation, and insulin resistance. When STAMP-2 is activated, the production of proinflammatory cytokines is inhibited through downregulation of nuclear factor kappa B {NFkB}, the key transcription factor for activation of genes responsible for the production of proinflammatory cytokines and enzymes such as IL-1-beta, TNF-alpha, COX2, and LOX5 [84]. STAMP-2 deficiency can lead to several disorders, including atherosclerosis, metabolic syndrome, and diabetes [85]. NFkB contributes to the development of insulin resistance, and STAMP-2 restores insulin sensitivity by inhibiting NFkB [86]. All members of the STAMP family possess both ferric and cupric reducing activities, which indicate that STAMP-2 might regulate iron or copper entry into cells [87]. STAMP-2 requires iron or copper as cofactors, suggesting that STAMP-2 is needed to maintain metabolic homeostasis. STAMP-2 further protects against atherosclerosis and stabilizes plaques in diabetic mice [88]. Decreased activity of STAMP-2 and similar proteins creates a signalling bottleneck that leads to insulin resistance [86, 88]. Lactoferrin is an “iron” carrying protein, which could explain its activating function on STAMP-2 [89].

Considering all these factors, it seems plausible that preprandial (before meal intake) exercise induces the production of anti-inflammatory myokines and that these myokines stimulate neutrophils to produce lactoferrin [89]. The release of lactoferrin into the circulation activates visceral adipocytes, which subsequently react with increased expression of STAMP-2 [83]. STAMP-2, in turn, inhibits NFkB and Janus kinase (JNK) activation and prevents the inhibition of insulin signalling.

## 6. Physical Activity Was Spontaneous during Evolution and Causes a Phenotypical Shift of the Immune Response during Immune Challenges

That exercise prevents postprandial inflammation makes good sense from an evolutionary perspective. From 2 million years ago until approximately 200 years ago, common threats to human health included starvation, dehydration, predation, climate, accidents, violence, and infectious disease [90]. With the exception of infectious disease, all these threats induced spontaneous activity (SPA) through activation of dopaminergic neuroanatomic nuclei in the brain, including the ventral tegmentum and the striatum [75]. The major neuropeptide systems that have been studied relative to spontaneous physical activity include cholecystokinin, corticotropin-releasing hormone, neuromedin U, neuropeptide Y, leptin, agouti-related protein, orexins, and ghrelin. All these systems influence dopaminergic signalling [91]. SPA stimulates the production of anti-inflammatory myokines by energy depletion of the contracting muscles. As mentioned earlier, these myokines drive neutrophils to produce antimicrobial/anti-inflammatory molecules {e.g., lactoferrin} and STAMP-2 in adipocytes and cells of the innate immune system, thus preventing overactivation of NFkB and the subsequent proinflammatory/insulin resistance response.

The cascade of neurochemical reactions in the brain, when faced with old danger factors such as starvation, prompted patterns of SPA that reflected the daily lives of our ancestors. To starve off hunger and obtain food, both men and women often engaged in fishing, while men also engaged in leg-based long-term hunting and women employed arm-based gathering. Our ancestor's use of the upper body during physical activity is an important factor in stimulating an anti-inflammatory reaction. Upper body muscles are energetically more efficient than lower body muscles and are normally conserved even during severe metabolic conflicts, as observed in people suffering from chronic obstructive pulmonary disorders (COPD) [92, 93]. Preservation of upper body muscles may seem counterintuitive considering that scientists unequivocally recognize horizontal running as the evolutionarily conserved flight direction and considering that the maximum running speed of predators such as lions, tigers, and jaguars is by far much higher than the maximum speed of the fastest human.

Conceivably, conserving upper body muscles during metabolically stressful situations may have occurred because humans could more easily escape threats by climbing/fighting than by running, making maintenance of the upper body muscles a priority. This view is supported by the observation that energy is first allocated to arm muscles during fear situations, manifesting in warm, sweating hands and an instantaneous increase of circulation [94–96]. Palm sweating has a surprising benefit; hands are important parts of the body to eliminate excessive body heat during exercise and increased heat elimination through palm sweating augments exercise resistance and prevents fatigue. Use of the upper body is also likely to have led to optimal levels of IL-6, which is anti-inflammatory only when produced in small amounts. These amounts depend on the amount of metabolic stress on contracting muscles during exercise, which is significantly less when using the upper body for fishing, digging, and other gathering functions. Our ancestors were merely gatherers/fishers and part-time hunters, and they therefore often relied on their upper body, thus producing the optimal anti-inflammatory cocktail of myokines to cause an anti-inflammatory postprandial response.

As a result, although our ancestors suffered from stress, these patterns of activity made them less likely than most modern humans to experience a metabolically expensive inflammatory response of the innate immune system and subsequent low-grade inflammation-based disorders. The energy thereby conserved would have been used to feed the metabolically expensive brain, and the absence of stress-induced insulin resistance would prevent the development of neurodegenerative diseases and other maladies affecting the central nervous system in general, but especially the brain [97].

## 7. Spontaneous Physical Activity and the Immune System

Evidence that SPA is caused by ancient stress factors such as food scarceness comes from research with people suffering from anorexia nervosa and individuals with obesity, that is, the opposite phenotype [74]. However, SPA and even hyperactivity in periods of reduced food availability are not unique to humans: animals need to be active to search for more food if the food supply is limited, as is commonly the case in the natural habitat. Already in 1922, the psychologist Curt Richter observed that if food is served for a limited period of time, the meal is preceded by an increase in physical activity [98]. This phenomenon is referred to as “food anticipatory activity” {FAA}, and the biological explanation for FAA preceding meals is similar to the above-mentioned evolutionary approach to human SPA in starvation.

The SPA pathway, activated through food restriction, seems to be dependent on orexin and dopamine [74]. Activation of orexin receptors leads to an increase in physical activity, while orexin is released in response to food restriction [99]. Orexin-producing hypothalamic neurons project on dopaminergic neurons in the ventral tegmentum, which are highly involved in SPA [100]. The same is true for the gut-derived orexigenic hormone ghrelin, which also activates dopaminergic pathways in the ventral tegmentum and induces SPA [101].

Dopaminergic pathways responsible for spontaneous physical activity have gained importance during evolution. These dopaminergic neurons belong to the group of the so-called emotional motor neurons [102–104] and these neurons are part of the behavioural column. The behavioural column and its emotional motor neurons literally facilitate muscle contraction through “gain setting” pathways and motivation. It can be concluded that motivated activity is energetically less costly (“cheap”) than voluntary exercise and motivated activity is dopamine dependent [105]. Comparison of the human substantia nigra and ventral tegmentum with those of other mammals or vertebrates revealed tremendous differences in the number of dopaminergic neurons. For example, lizards {*Gekko*} have about 2,000 dopaminergic neurons and turtles (*Pseudemys*) about 5,500, while rats have 45,000 dopaminergic neurons, macaques 165,000, and humans 590,000 in the first four decades of their lives [106]. The large number of dopaminergic neurons in humans is remarkable given that the human brain is only three times larger than that of the macaque. Normally, the number of dopaminergic neurons correlates closely with body weight, but humans are a clear exception.

This peculiarity is even more striking because a single dopaminergic neuron of the substantia nigra or ventral tegmental area (VTA) sends many collaterals with large arborization trees to several areas of the forebrain. The projections of these neurons are essentially similar among the different vertebrate species, though they tend to abundantly innervate the striatal areas and, less abundantly, pallial {cortical} areas, underscoring their role in the control of incentive and sensorimotor behaviours. Details of these connections are well described only in mammals and especially in humans [107]. As mentioned before, the dopaminergic motor system belongs to the behavioural column defined by Swanson [103], and this system facilitates and permits behaviour [51]. The opposite response is observed in people with a variety of diseases such as fibromyalgia syndrome, multiple sclerosis, panic disorder, obesity, and Parkinson's disease, all of whom show nonpermissive behaviour such as exercise avoidance [108]. Dopamine-induced preprandial SPA firstly saves energy for brain function and secondly helps to induce a protective anti-inflammatory response of the immune system after food intake through upregulation of the production of lactoferrin and its accumulation in dopaminergic neurons might protect against neurodegeneration and Parkinson's disease [109].

As mentioned before, several specific aspects of human locomotion have made it possible to save energy for the brain [23], but this is not the only way the brain gained access to more resources to grow and develop during hominin evolution. Chronic exercise shaped the immune system, creating a highly protective anti-inflammatory phenotype that maintains protection against possibly lethal invaders while saving energy [82].

## 8. Physical Activity: Part of the Combined and Coordinated Neuroendocrine-Immunological Stress Response

Another intriguing effect of spontaneous or voluntary exercise is related to its ability to convert dopamine to codeine and morphine, although in low amounts [110]. Codeine and morphine are known for their analgesic effects, but much less is known about their influence on the immune system. Endogenous codeine and morphine induce constitutive nitric oxide synthetase (cNOS) in immune cells. The resulting NO inhibits mitochondrial energy (ATP) production in white blood cells and thereby immune inflammatory activity [111]. Voluntary motivated exercise also produces the immunoregulatory/anti-inflammatory cytokine IL-10, which may further elicit anti-inflammatory effects through dopamine-regulated physical activity [112, 113]. This anti-inflammatory response might be dangerous when the bacterial load is so high that the host would be in serious infectious danger when the immune system would maintain an anti-inflammatory activity. It is in this context not surprising that recent research showed that when the pathogenic load is high, the immune response will maintain proinflammatory capacity and drive sickness behaviour and thereby outweigh the anti-inflammatory potential of voluntary exercise [114]. This observation in mice further supports the selfish character of the immune system. Taken together, both the emergence and function of dopamine, morphine, and nitric oxide seem to be related to evolutionary pressures to maintain chronic physical activity (for an excellent review see [115]). The combined production of lactoferrin, anti-inflammatory myokines, and IL-10 and also the dopamine/morphine/NO triad through motivated and spontaneous exercise make it at least plausible that SPA has driven the immune system to an anti-inflammatory phenotype, thereby saving energy for the brain and the muscles, which is in support of our hypothesis and observed mechanisms.

## 9. Physical Activity Protects against the Damaging Effect of the Proinflammatory Activity of the Selfish Immune System: Clinical Evidence

Clinical research in patients suffering from different disorders of the central nervous system supports the notion that physical activity protects against proinflammatory activity. Patients with neuroinflammatory diseases such as amyotrophic lateral sclerosis (ALS) react positively to exercise [116], probably through downregulation of the immune response directed against brain tissue. Patients with Parkinson's disease who engage in “forced” exercise show significant improvement of typical Parkinson symptomatology and less progression of the disease [117]. These and other neurodegenerative and many other diseases seem to be related to disturbances in energy metabolism in the brain [118] and also to energy distribution between the central nervous system and the peripheral organs [119], all caused, at least in part, by a chronically activated immune system [10, 120].

In summary, SPA and recreational voluntary exercise seems to override the chronically active immune system [121] and reduce its selfish behaviour. Strenuous obligatory exercise produces immune suppression, possibly leading to increased susceptibility to infection [114, 122], although recent human research shows that the immune system reactivates when challenged with a high load of pathogens in people engaging in strenuous exercise. Physical activity {ancestors = SPA, contemporary = voluntary} induces anti-inflammatory myokines which inform the immune system and literally relax it. A relaxed immune system (which is distinct from “suppressed”) only reacts to the danger signals it was designed to respond to, such as bacteria and viruses, which conserves energy for the benefit of phylogenetically younger organs such as the liver, kidneys, and brain and gives rise to normal behaviour and a so-called permissive brain phenotype. A subject's permissive brain literally facilitates free will, as opposed to those suffering from a nonpermissive brain syndrome [51, 123–126]. Behavioural changes have high energetic cost and lack of brain energy prevents flexibility and loss of free will. Spontaneous and voluntary activity can also be considered a strategy belonging to the proactive behavioural immune system. Avoiding danger, psychoemotional problems, social contact with infected individuals, dirt, and other unknown, possibly dangerous, triggers relax the expensive reactive immune system, once again providing energy for the human brain. Recent research in insects shows that an acute flight/fight reconfigures the type of immune reaction from a pro- to an anti-inflammatory phenotype, just as in humans. Once more, it is this low-cost anti-inflammatory immunological response that protects against infection and saves energy for brain development and function. In contrast, the sedentary lifestyle, a disease in itself, demands a proinflammatory response of the immune system and chronic allocation of energy/resources to this system. Low-grade inflammation is the ultimate consequence and the cause of causes of most, if not all, noncommunicable chronic diseases [46].

## 10. Conclusions

Because regular muscle contraction diminishes the need for proinflammatory activity of the innate immune system [127], we suggest that muscles should be considered part of the immune system [128]. The phylogenetic development of muscles, together with that of human locomotion, increased fat storage. Differences in glucose transporters between muscle and the brain have been responsible for patterns of energy allocation that enabled the brain to grow to its evolutionary maximum of 1,490 mL approximately 30,000 years ago. Throughout hominin evolution, our ancestors encountered dangerous situations, such as violence and dehydration, that threatened the survival of the individual or the species and usually trigger a survival response in which more ancient systems tend to dominate younger ones [129], although brain functions and anatomy seem to maintain the highest priority. The normal response to survival threats is SPA, and SPA is capable of controlling the immune system by saving energy for the brain during stressful situations [130]. The absence of SPA or voluntary exercise, as is occurring in sedentary people living modern lifestyles, provides a framework in which the immune system overrides the interests of the normally selfish brain, which could be the reason why brain size shrank in the last 30,000 years from the maximum of 1,490 mL to the current average of 1,350 mL.
